# miRNA-Mediated Functional Changes through Co-Regulating Function Related Genes

**DOI:** 10.1371/journal.pone.0013558

**Published:** 2010-10-22

**Authors:** Jie He, Jin-fang Zhang, Can Yi, Qing Lv, Wei-dong Xie, Jian-na Li, Gang Wan, Kai Cui, Hsiang-fu Kung, Jennifer Yang, Burton B. Yang, Yaou Zhang

**Affiliations:** 1 School of Life Sciences, Tsinghua University, Beijing, People's Republic of China; 2 Division of Life Science, Graduate School at Shenzhen, Tsinghua University, Shenzhen, People's Republic of China; 3 Li Ka Shing Institute of Health Sciences, The Chinese University of Hong Kong, Hong Kong, People's Republic of China; 4 Sunnybrook Health Sciences Centre, Department of Laboratory Medicine and Pathology, University of Toronto, Toronto, Canada; 5 Department of Physiology, University of Toronto, Toronto, Canada; George Mason University, United States of America

## Abstract

**Background:**

microRNAs play important roles in various biological processes involving fairly complex mechanism. Analysis of genome-wide miRNA microarray demonstrate that a single miRNA can regulate hundreds of genes, but the regulative extent on most individual genes is surprisingly mild so that it is difficult to understand how a miRNA provokes detectable functional changes with such mild regulation.

**Results:**

To explore the internal mechanism of miRNA-mediated regulation, we re-analyzed the data collected from genome-wide miRNA microarray with bioinformatics assay, and found that the transfection of miR-181b and miR-34a in Hela and HCT-116 tumor cells regulated large numbers of genes, among which, the genes related to cell growth and cell death demonstrated high Enrichment scores, suggesting that these miRNAs may be important in cell growth and cell death. MiR-181b induced changes in protein expression of most genes that were seemingly related to enhancing cell growth and decreasing cell death, while miR-34a mediated contrary changes of gene expression. Cell growth assays further confirmed this finding. In further study on miR-20b-mediated osteogenesis in hMSCs, miR-20b was found to enhance osteogenesis by activating BMPs/Runx2 signaling pathway in several stages by co-repressing of PPARγ, Bambi and Crim1.

**Conclusions:**

With its multi-target characteristics, miR-181b, miR-34a and miR-20b provoked detectable functional changes by co-regulating functionally-related gene groups or several genes in the same signaling pathway, and thus mild regulation from individual miRNA targeting genes could have contributed to an additive effect. This might also be one of the modes of miRNA-mediated gene regulation.

## Introduction

miRNAs are a class of small non-coding RNAs, which play pivotal roles in various biological processes including cell fate choices of embryonic stem cells, cell proliferation, apoptosis, development, differentiation, morphogenesis, carcinogenesis and angiogenesis[3]–[14]. Mature miRNAs repress gene expression post-transcriptionally by binding to regulatory targets in the 3-untranslated regions (3′UTRs) of mRNAs, then leading to translational repression or mRNA degradation [3], [15]. Some different action modes were also reported in a few mRNAs, including transcriptionally inducing or silencing gene expression through binding to target sites in the promoter region of a gene [16], [17]. Recently, Eiring et al. reported that miR-328 can act as a decoy by binding to a regulatory RNA binding protein and preventing it from blocking translation of mRNAs [18]. However, post-transcriptionally repressing gene expression by binding to 3′UTRs is still the mainstream regulative mode of miRNAs and the complexity of this mode is gradually emerging.

Computational prediction and biologic data from investigations of genome-wide scale show that one miRNA may target tens to hundreds of genes [19]–[21]. Recently data gathered using a proteomic approach also demonstrated that a single miRNA can repress the production of hundreds of proteins, but the extent of miRNA-mediated repression is surprisingly mild[1], [2], so it is difficult to understand how a single miRNA is able to provoke a detectable functional change, with such a mild regulation. This widespread, often subtle and customized, influence of miRNAs on mRNA expression was introduced by Bartel and Chen as the ‘micromanager model’ or ‘tuning’ of miRNA function [22]. “Tuning” gene's expression to keep balance might be one of the important functions of miRNAs, however, it seems hard to reconcile with the reported critical functions of miRNAs in a broad range of biological and pathological processes. Seitz proposed that miRNAs can not fine-tune many targets, because many computationally identified miRNA targets may actually be competitive inhibitors of miRNA function, preventing miRNAs from binding their authentic targets by sequestering them. miRNAs would rather repress only a few authentic targets, but those targets would be repressed sufficiently for that regulation to have a physiological effect [23]. Here, we propose that fine-tuning target expression can have a large effect, by co-regulating functionnally related genes under certain conditions. However, evidences are needed to support this hypothesis.

In this study, we re-analyzed the data from investigations of genome-wide scale by employing a bioinformatic assay[1], [24], and found that the transfection of a single miR-181b or miR-34a in Hela or HCT 116 tumor cells regulated large numbers of genes. Among them, the genes related to cell growth and cell death displayed high Enrichment scores, suggesting that these two miRNAs may be involved in cell growth and cell death, and that was confirmed using biological assays. In our further study on miR-20b mediated osteogenesis in hMSCs, we found that miR-20b enhances osteogenesis by activating several levels of the BMPs/Runx2 signaling pathway, which includes co-repressing PPARγ, Bambi and Crim1. Our data suggests that miR-181b, miR-34a and miR-20b co-regulate a group of functionally related genes to provoke detectable functional changes under certain condition.

## Results and Discussion

### miR-181b and miR-31a co-regulate large numbers of functionally related genes to cause functional changes of the cells

In order to investigate if there is a co-regulative pattern in miRNA-mediated functional change, we studied the effect of miR-181b and miR-31a on cell growth and cell death. First, we re-analyzed the biologic data from investigations of genome-wide scale [1], [24], using a bioinformatics program called DAVID [25] and grouped miRNA regulated genes into functional annotation clusters. If co-regulative pattern plays a role in miR-181b- and miR-34a-mediated cell growth or death, the gene clusters related to cell growth and cell death would have high Enrichment scores. According to the study by Lempicki and coworkers, a higher Enrichment score for a cluster indicates that the gene members in the cluster are involved in more important (enriched) terms in a given study [25].

Bartel and coworkers used quantitative mass spectrometry to measure the response of thousands of proteins after introducing miR-181b into Hela cells [1]. We analyzed their proteomic data with DAVID bioinformatics resources and found that a total of 674 genes were regulated by miR-181b when cut off value was ±0.2 (log_2_-fold-change), corresponding to a 15% change. We uploaded all of these genes into DAVID bioinformatics resources for functional annotation clustering. The result showed that five clusters are related to cell growth and cell death, and their Enrichment scores are 4.1, 2.23, 2.22, 1.79 and 1.64, all higher than 1.3 (Table S1). An enrichment score of 1.3 is equivalent to a non-log scale value of 0.05. More attention should be given to groups with scores ≥1.3 [25]. These results suggest that miR-181b may regulate cell growth and cell death.

After removing the overlapping genes among the five clusters, we found 153 genes related to cell growth and cell death (Table S2). Among them, 86 genes are down regulated and 67 genes are up regulated; 109 genes (N+P) are reported positively or negatively regulating cell growth or death and the other 44 (D) may have dual effects or unclear effects on cell activities (Table 1 and Table S2). Among 76 genes that have been previously determined to stimulate cell growth or inhibit cell death, 42 genes were up-regulated (Table 1). On the other hand, 25 out of 33 genes that can inhibit cell growth or promote apoptosis were down regulated. Thus, miR-181b induced a change in protein expression of 67 out of 109 (61.5%) genes related to enhancing cell growth and decreasing cell death. Our data suggests that miR-181b may stimulate Hela cell growth and inhibit cell death. However the exact function of this miRNA on cell growth depends on the comprehensive effect of miR-181b regulated genes, and not on the number of miRNA regulated genes that are predicted to activate cell growth or inhibit cell death, so thus biological assays should be used to confirm the exact roles of miR-181b in Hela cells.

**Table 1:** Effect miR-181b induced genes on cell growth and cell death.

Effect on cell growth or cell death	Up-regulated genes	Down-regulated genes	Total
P	42 (a)	34 (b)	76
N	8 (c)	25 (d)	33
D	17 (e)	27 (f)	44
total	67	86	153

P: stimulating cell growth or inhibiting cell death;

N: inhibiting cell growth or enhancing cell death;

D: dual effects or unclear effects on cell growth or cell death.

**Figure 1:**
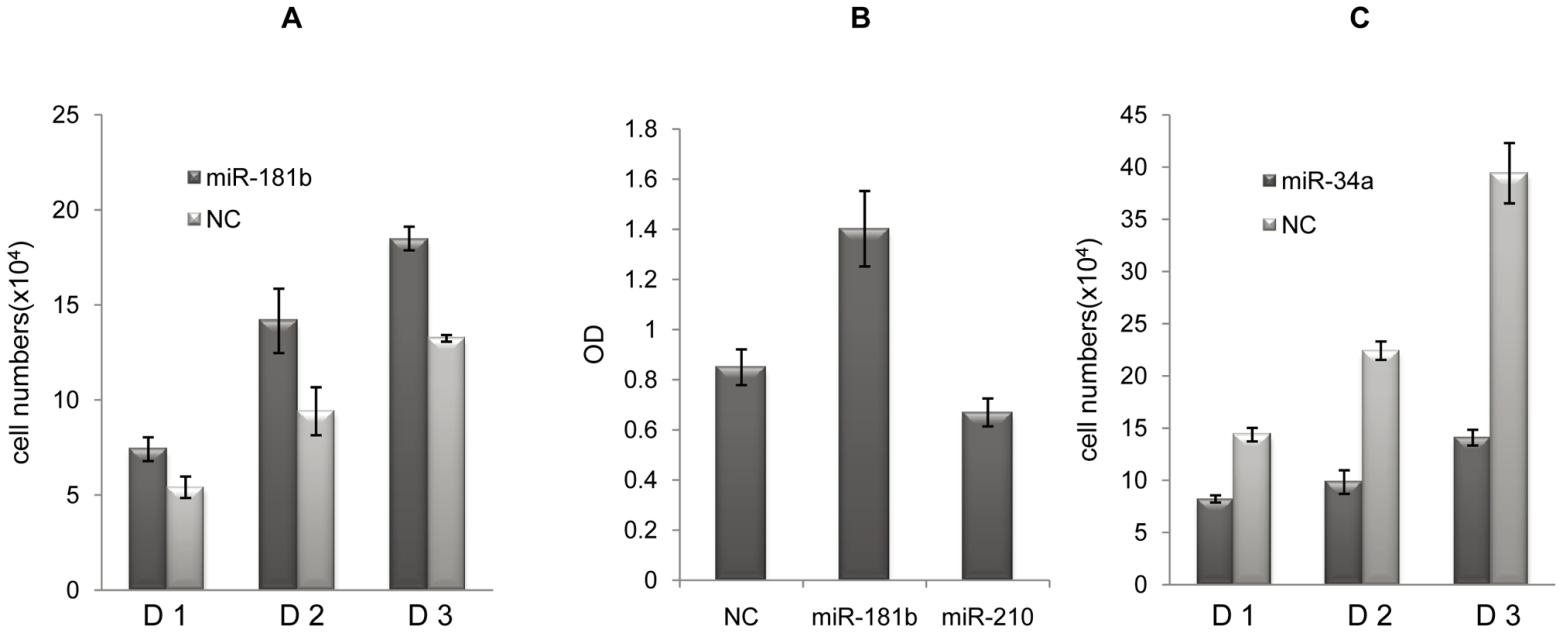
Cell growth assay of miR-181b- and miR-34a-transfected tumor cells. Hela cells (A), CNE cells (B) and HCT 116 cells (C) were transfected with the mimics of miR-181b, miR-34a and miR-210. Small RNA with random sequence (NC) was used as a control. Cells were cultured for three days and cell numbers were counted every day (A and C) or SRB (sulforhodamin B) was used for cell growth assay (B).

In order to confirm the role of miR-181b in cell growth, we transfected Hela cells with the mimic of miR-181b and evaluated the effect of miR-181b transfection on cell growth and death, a mimic with random sequence (NC) was used as control. The cells transfected with miR-181b grew faster than the control (Fig. 1A). We also studied the role of miR-181b in inducing cell growth in CNE cells, a nasopharyngeal carcinoma cell line (Fig. 1B).

Nakagama and co-workers reported that miR-34a caused complete suppression of cell proliferation and apoptosis in HCT 116 cells. They transiently introduced miR-34a into two human colon cancer cell lines, HCT 116 and RKO for gene-expression microarray assay [18]. We analyzed the biologic data from miR-34a-transfected HCT 116 cells with the DAVID bioinformatics resources [25] and found that 451 genes were regulated by miR-34a when the value of change in gene expression was cut off at 2 folds. Functional annotation clustering then demonstrated that six clusters were related to cell growth and cell death, and that the Enrichment scores of these clusters are 5.77, 2.62, 1.91, 1.71, 1.52 and 1.35 (Table S3). Among 451 miR-34a-regulated genes, 120 of them are related to cell cycle or cell death (after removing the overlapping genes). Among them, 59 genes were down regulated and 61 genes were up regulated through direct or indirect interaction with miR-34a (Table S4). With the exception of 36 genes that have been suggested to exert dual effects or unclear effects on cell growth or cell death (D), 85 genes (N+P) were to positively or negatively affect cell growth or cell death (Table 2 and Table S4). Amongst those 85, miR-34a altered the gene expression of 52 of them (61.2%) all related to inhibiting cell growth and enhancing apoptosis. This data suggests that miR-34a could potentially inhibit cell growth and enhance apoptosis of HCT-116 cells. Since the comprehensive effect of the miRNA on cell growth also depends on the quantitative effect of each of those miR-34a regulated genes on cell growth, biological assays are needed to confirm the exact role of miR-34a on HCT-116 cells. Nakagama's findings [18] and the results from our investigation are consistent with what was found in the bio-informatics assay, all demonstrating that miR-34a can inhibit HCT-116 cell growth and enhance apoptosis (Fig. 1C).

**Table 2:** Effect miR-34a induced genes on cell growth and cell death.

Effect on cell growth or cell death	Up-regulated genes	Down-regulated genes	Total
P	20 (a)	31 (b)	51
N	21 (c)	13 (d)	34
D	20 (e)	15 (f)	35
total	61	59	120

P: stimulating cell growth or inhibiting cell death;

N: inhibiting cell growth or enhancing cell death;

D: dual effects or unclear effects on cell growth or cell death.

Taken together, miR-181b and miR-34a were able to cause an obvious change in cell proliferation and/or apoptosis by co-regulating a large number of cell growth- and cell death-related genes, which together, exerted an accumulative effect. However, an issue with transfecting miRNA in cultured cells is that they can produce excessive miRNA concentrations that are far greater than the physiological concentrations found in biological settings. As a result, changes in cell physiology or morphology due miRNA transfection may be different from the effects caused by an endogenous miRNA.

### miR-20b co-regulates several genes in the same signaling pathway to generate functional changes of the cells

In previous experiments, we found that miR-20b can enhance osteogenesis in human mesenchymal stem cells (hMSCs). Since BMP/Runx2 signaling is critical in regulating osteoblast differentiation [26], in this investigation, we studied if and how miR-20b caused significant change in MSC differentiation by co-regulating various genes in the BMPs/Runx2 signalingpathway to accumulate the mild regulation from individual miRNA target genes and produce an accumulative effect as a whole.

**Figure 2:**
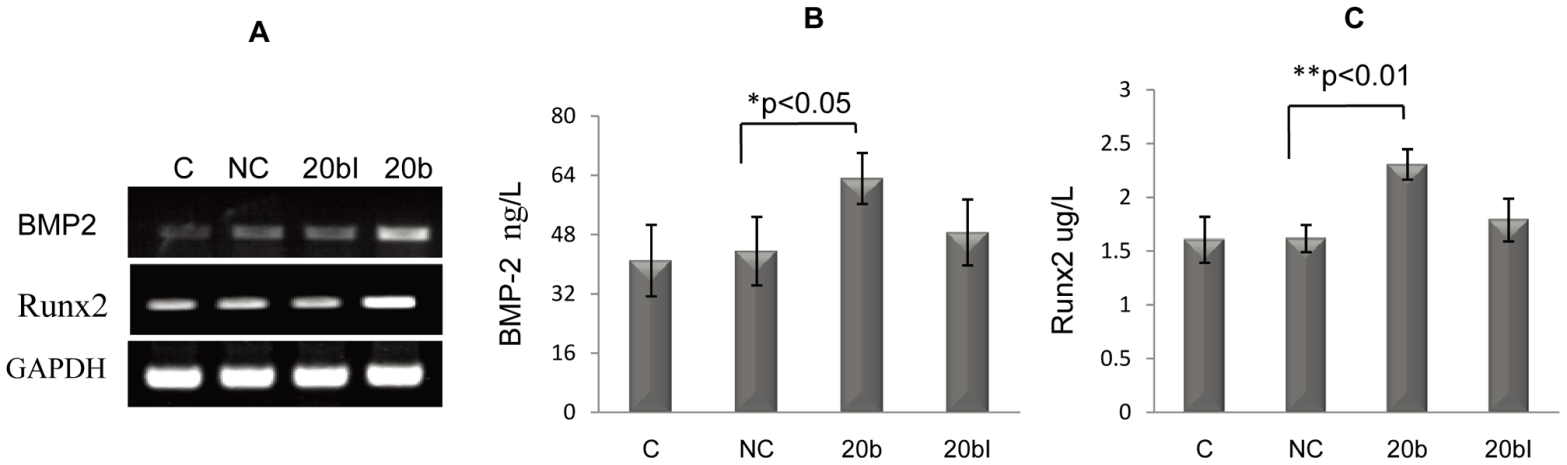
miR-20b activates BMP/Runx2 signaling pathway. (A) miR-20b mimic-transfected MSCs were collected at day 6. RT-PCR results showed an up-regulation on the mRNA expression of BMP2 and Runx2 by miR-20b at day 6 in hMSCs respectively. (B) and (C) are the results of ELISA analysis on the expression of BMP-2 and Runx2 on day 6 during osteogenesis after miR-20b transfection (n = 4, *p<0.05, **p<0.01.) C: control; NC: negative control (small RNA with random sequence); 20bI: miR-20b inhibitor.

As revealed by RT-PCR and ELISA, transfection of miR-20b into hMSCs significantly elevated expression levels of BMP-2 and Runx2 both at the mRNA and the protein levels (Fig. 2). Usually, miRNAs negatively regulate gene expression. Therefore, it is possible that miR-20b up-regulates the expression of both BMP2 and Runx2 by suppressing their negative regulators or inhibitors. Among the negative regulators or inhibitors of BMP/Runx2 signaling pathway, PPAR_γ_, the most important cell fate determining factor in MSC differentiation, is the best candidate, because it positively regulates adipogenesis but negatively regulates osteogenesis by inhibiting the transcription of Runx2 as well as by down-regulating BMPs expression [27]–[30]. Thus, we investigated whether or not PPAR_γ_ is a target gene of miR-20b.

**Figure 3:**
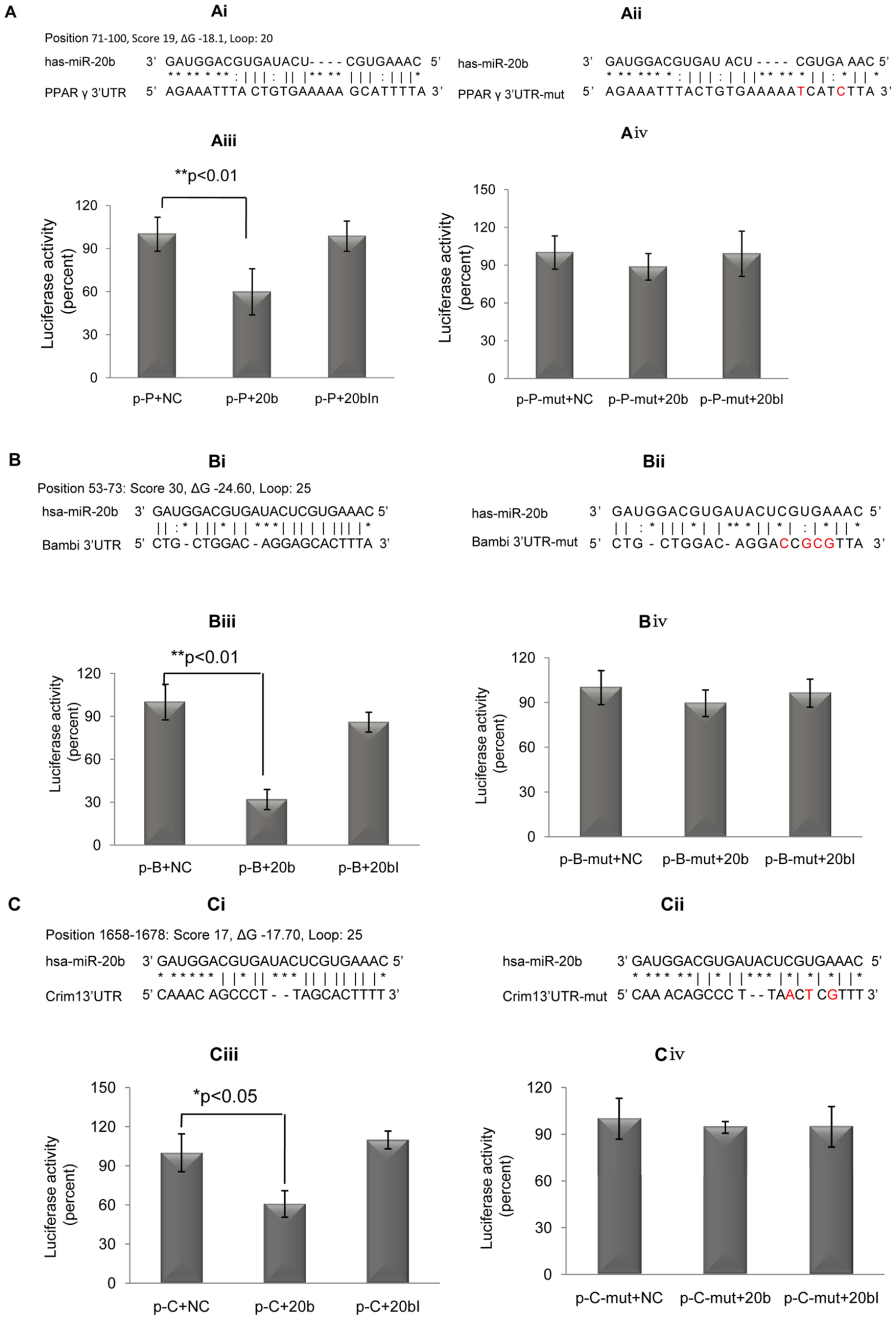
miR-20b activates BMP/Runx2 signaling pathway by down-regulating the expression of PPARγ, Bambi and Crim1. A. Predicted (Ai) and mutated (Aii) miR-20b binding sites in the 3′UTR of PPARγ. (Aiii) luciferase activity of the pRL-TK-PPARγ report vector was inhibited by miR-20b and the mutation on the binding sites of the 3′UTR of PPARγ eliminated the repressive effect of miR-20b (Aiv) (n = 6; *p<0.05). p-p+NC: pRL-TK-PPARγ+ negative control; p-p+20b: pRL-TK-PPARγ + miR-20b; p-p+20bI: pRL-TK-PPARγ + miR-20b inhibitor. p-p-mut+NC: pRL-TK-PPARγ mutation plasmid+ negative control; p-p-mut+20b: pRL-TK-PPARγ mutation plasmid+miR-20b; p-p-mut+20bI: pRL-TK-PPARγ mutation plasmid+miR-20b inhibitor. B. Prediction (Bi) and mutation (Bii) of miR-20b binding sites in the 3′UTRs of Bambi. (Biii) Luciferase activities of report vector with the 3′UTR of Bambi were inhibited by miR-20b and the mutation on the binding sites of the 3′UTR of Bambi (Biv) eliminated the repressive effect of miR-20b (n = 6; *p<0.05). C. Prediction (Ci) and mutation (Cii) of miR-20b binding sites in the 3′UTRs of Crim1. (Ciii) Luciferase activities of report vector with the 3′UTR of Crim1 were inhibited by miR-20b and the mutation on the binding sites of the 3′UTR of Crim1 (Civ) eliminated the repressive effect of miR-20b (n = 6; **p<0.01). *p-B/p-C+NC*: pRL-TK-Bambi/pRL-TK-Crim1 plasmid + negative control; *p-B/p-C+20b*: pRL-TK-Bambi/pRL-TK-Crim1 plasmid + miR-20b; *p-B/p-C+20bI:* pRL-TK-Bambi/pRL-TK-Crim1 plasmid + miR-20b inhibitor. *p-B-mut/p-C-mut+NC:* pRL-TK-Bambi/pRL-TK-Crim1mutation plasmid + negative control; *p-B-mut/p-C-mut+20b:* pRL-TK-Bambi /pRL-TK- Crim1 mutation plasmid + miR-20b; *p-B-mut/p-C-mut+20bI:* pRL-TK-Bambi/pRL-TK- Crim1 mutation plasmid + miR-20b inhibitor.

We did so by using *FindTar*, a computational algorithm developed in our Lab (http://bio.sz.tsinghua.edu.cn/lab/findtar) [31], and found that miR-20b consisted of a highly probable binding site at the 3′UTR of PPAR_γ_ (Fig. 3Ai). We then further investigated the interaction between the 3′UTR of PPAR_γ_ and miR-20b. The 3′UTR of PPAR_γ_ was inserted into a luciferase expression vector to generate a luciferase reporter construct. The miR-20b was then co-transfected with this reporter construct into Cos-7 cells and the levels of luciferase activity were measured to determine the corresponding repressive effects. Indeed, we detected a significant repression in luciferase activity when miR-20b was co-transfected with this vector expressing the 3′UTR of PPAR_γ_ (Fig. 3Aii). To further confirm this direct interaction, the binding site in PPAR_γ_ 3′UTR was mutated to generate another reporter vector (Fig. 3Aiii). Such a mutation abolished the repressive effects that miR-20b had on luciferase activity (Fig. 3Ai), suggesting that miR-20b regulates PPAR_γ_ expression by directly binding to the 3′UTR site of PPAR_γ_.

During the search for target genes of miR-20b by employing such bioinformatics softwares as miRanda [32], Pictar [19], TargetScan [33] and Findtar [31], two antagonists of the BMP pathway, Bambi and Crim1, were also recognized as putative target genes. To deduce the relationships between these two antagonists and miR-20b, the 3′UTR (i.e. 237 bp) of human Bambi or the 3′UTR (i.e. 174 bp) of human Crim1 were cloned into the pRL-TK vector. As seen in Fig. 3Bi and Ci, both the 3′UTRs contain putative binding sites for miR-20b. Cos-7 cells were cotransfected with the vectors, negative control and miR-20b, respectively. Cotransfection of miR-20b caused significant reduction in total luciferase activity (Fig. 3Biii and Ciii). To further demonstrate that an interaction exists between this miRNA and Bambi or Crim1, the same 3′UTR of Bambi or Crim1 was mutated and cloned into the pRL-TK vector. Figure 3Bii and Figure 3Cii shows the mutation we created on the binding sites of the 3′UTR of both Bambi and Crim1. Based on our results, this mutation eliminated the repressive effect that miR-20b had on luciferase activity(Fig. 3Bi and Ci). These results indicate that miR-20b may directly target the 3′UTRs of both Bambi and Crim1, thus repressing their expression. Bambi is a pseudo-receptor of BMP while Crim1 is an essential factor that tethers growth factors including BMPs at the cell surface [30], [34]–[36]. The down-regulation of Bambi by miR-20b could perhaps free up more BMP molecules that functional BMP receptors can then bind to, thereby increasing the expression of Runx2. Suppression of Crim1 observed after the transfection of miR-20b resulted in an increase in effective BMP concentrations in the media, which also affected BMP processing and delivery to the cell surface. It has been postulated that such an increase in free, mature and active BMPs could activate BMP signaling, and thus enhance osteogenesis of MSCs as demonstrated in our study.

**Figure 4:**
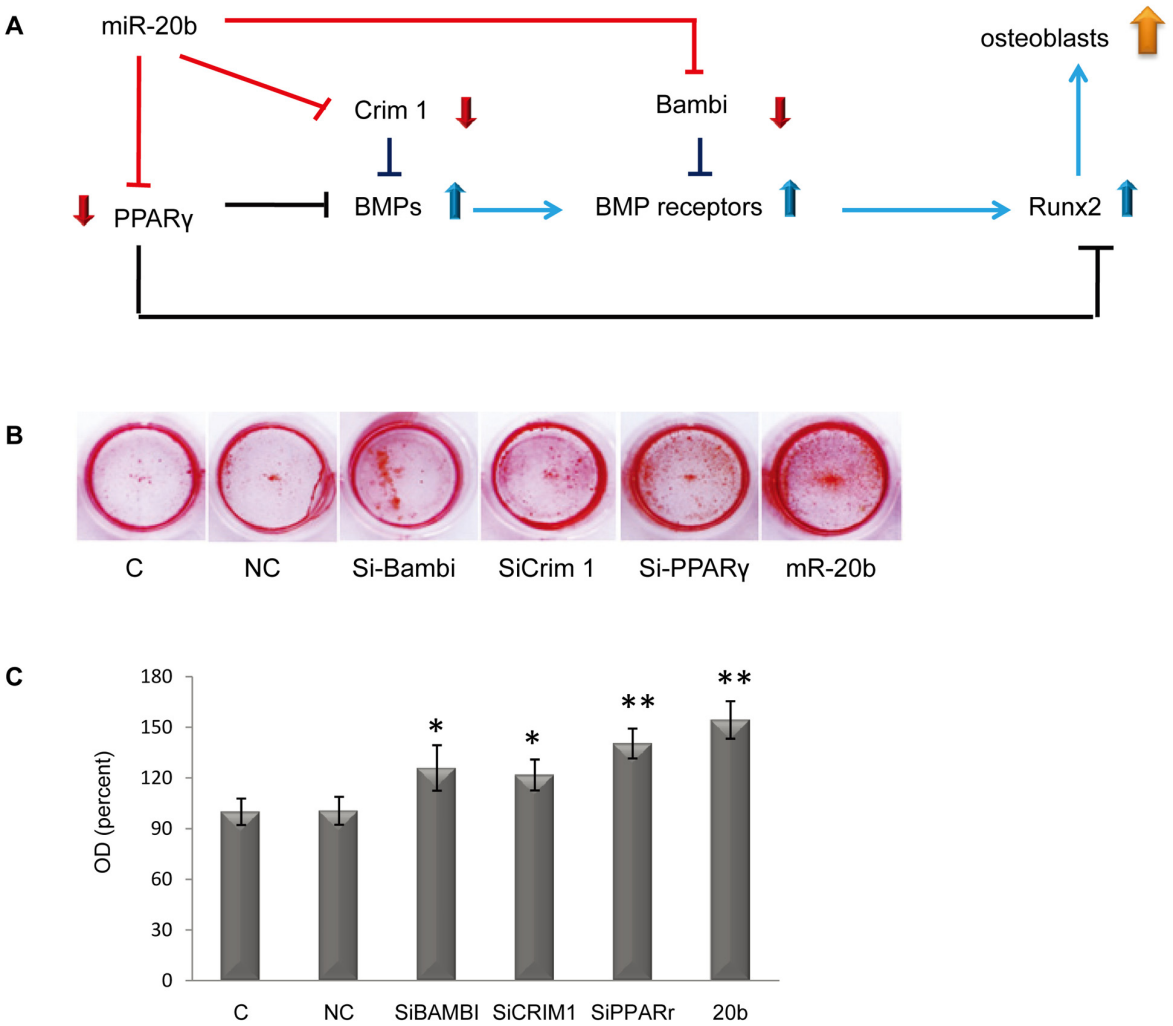
miR-20b targeting several repressive genes in the BMPs/Runx2 pathway induces miR-20b-mediated osteogenesis in hMSCs. (A) miR-20b enhanced osteogenesis of hMSCs by repressing the expression of inhibitors of the BMP/Runx2 signaling pathway in different levels. (B) Comparison of ARS staining between miR-20b-transfected hMSCs and siRNA-transfected hMSCs (designed to target miR-20b's target genes, PPARr, Bambi and Crami). (C) Des-staining and OD measurement of ARS staining samples.

In this study, we found that miR-20b enhanced osteogenesis by activating the BMPs/Runx2 signaling pathway at four levels, which consists of repressing PPAR_γ,_ Bambi and Crim1(Fig. 4A). However, we are curious whether or not miR-20b's ability to enhance osteogenesis is comparable to that of siRNAs of miR-20b's individual target genes in the BMPs/Runx2 signaling pathway. Thus, we transfected hMSCs with the mimic of miR-20b and the siRNAs for Alizarin red S staining (ARS staining). The experiment showed that miR-20b induced a stronger change in ARS staining than the siRNA (Figure 4B and 4C). This might be due to the multi-target characteristics of miR-20b, which siRNAs do not possess.

Taken together, miR-181b, miR-34a and miR-20b may have provoked detectable functional changes by co-regulating functionlly related gene groups or several genes in the same signaling pathway. The accumulation of mild regulation from individual miRNA targeting genes could have then lead to a detectable change. This appears to be one of the modes of miRNA mediated gene regulation.

## Materials and Methods

### Ethics statement

Human MSC was isolated from bone marrow collected from an eighteen-year old healthy female donor under written consent by the patient and under approval by The Chinese University of Hong Kong- New Territories East Cluster Clinical Research Ethics Committee.

### Bioinformatic assay

The biologic data from investigations of genome-wide scale of Bartels' proteomic assay [1] and Nakagamas' gene-expression microarray assay [24] were analyzed for functional annotation clustering, using the David tool (http://david.abcc.ncifcrf.gov/). All significantly enriched GO terms (Benjamini-Hochberg corrected p-values <0.05) are shown in the Table S1 and Table S3.

### Cell Growth and Death Assay

Hela cells (ATCC, VA), HCT116(IBMS of CAMS, China) cells, and CNE cells of a nasopharyngeal carcinoma cell line (Kunming Cell Bank, China) were seeded in 24-well culture plates (2×10^4^ cells /well) and cultured in Dulbecco's modified Eagle's medium (DMEM), Iscove's Modified Dulbecco's Medium (IMDM) and RPMI 1640 containing 10% fetal bovine serum (FBS) overnight, respectively. After miRNA transfection and serum starvation, the cells were continuously cultured in basal media with 2.0% FBS. After transfection, cells were harvested and counted every day for 3 days. The average number of cells was determined at each time point in triplicates.

### MSCs isolation, culture and induction of osteogenic differentiation

Bone marrow was collected from an eighteen-year old healthy female donor. hMSCs were isolated and identified by cell surface markers, then, cultured in Minimum Essential Medium Alpha Medium (α-MEM) supplemented with 10% FBS, 2 mM L-Glutamine, 100 U/mL penicillin and 100 µg/mL streptomycin, and incubated in a humidified atmosphere of 5% CO_2_ at 37°C. After four cell passages, cell surface markers, CD3, CD16, CD19, CD33, CD34, CD38, CD45, CD133, HLA-DR, CD29, CD44, CD90, CD105 and CD166 were assayed. Osteogenic differentiation of MSCs was induced according to a previously published protocol. OS medium was supplemented with osteogenic inducers, including 10^−8^ M dexamethasone (Dex), 10 µg/mL L-ascorbic acid 2-phosphate (AsAP) and 10 mM Glycerol 2-phosphate (Gly) (Sigma, USA).

### miRNAs transfection

miRNA mimics were designed according to the miRBase sequence database (http://microrna.sanger.ac.uk). miRNA mimics and siRNAs duplexes of PPARγ, Bambi, crim1 were synthesized by Shanghai GenePharma Co. (Shanghai, China). siRNA duplexes consisting of random sequence were used as negative controls (NC). The Table S5 shows the sequence of miRNA and siRNAs. Hela cells, CNE cells and hMSCs were seeded onto 96- or 24-well plates and transfected with siRNA/miRNAs duplexes using Lipofectaime 2000 (Invitrogen Corp., Carlsbad, CA) according to manufacturer's instructions. The medium was replaced 6 hours later.

### ARS staining

MSCs were fixed in 4% paraformaldehyde (m/v) for 10 min, and samples were evaluated by ARS. Briefly, cells were stained with 2% ARS (pH 4.1) for 15 min and then washed with deionized water twice. The orange and red positions in ARS staining were recognized as calcium deposits.

### Reverse transcriptase - polymerase chain reaction (RT-PCR)

hMSCs were harvested and the total RNA was extracted using Trizol reagent (Invitrogen, USA) according to the manufacturer's protocol. RT-PCR was performed with the total RNA using TaKaRa one step RNA PCR kit (TaKaRa Bio Inc, Japan). Primers were designed by the website: http://frodo.wi.mit.edu/based on the mRNA sequences obtained from Genebank (Table S6).

### Enzyme-linked immunosorbent assay (ELISA)

ELISA Kits from the R&D system (Minneapolis, MN) were used for ELISA assays of BMP-2 and Runx2. Briefly, the cell lysate of MSCs was added to the coated wells of a 96-well plate, then incubated with HRP-linked antibody at 37°C for 30 min after washing with washing buffer. Absorbance was determined at 450 nm on the microplate spectrophotometer (TECAN). Each experiment was done in quadruplets.

### Luciferase Assays

Reporter vectors were generated by inserting the 3′UTRs of Bambi (nt 14–250), Crim1 (nt 1584–1758) and PPAR_γ_ (nt 23–207) and their corresponding mutated 3′UTR fragments into pRL-TK plasmid (Promega, USA). 3′UTRs were amplified by PCR using the primers shows in Table S1. Luciferase assays were performed according to a previously published protocol [31]. Briefly, Cos-7 cells were seeded onto 24-well plates and cotransfection was performed using 300 ng of plasmid and 20 nm of each miRNA using Lipofectamine 2000, the next day. Cell lysates were collected after 30 h of transfection. Renilla luciferase activity were measured using a Luciferase Reporter Assay System (Promega, USA) and each experiment was repeated in three times. Total protein concentrations were determined at 595 nm using Bradford assay (Bio-Rad) on a spectrophotometer (TECAN). The luciferase activity was normalized by the total protein content.

### Statistical analysis

Data are expressed as mean ± SD. Statistical analysis was performed using the independent simplest-test (SPSS, USA). A *p*-value less than 0.05 was considered statistically significant.

## Supporting Information

Table S1 The functional annotation clusters related to cell growth and cell death in miR-181b regulated genes.
(0.24 MB DOC)

Table S2 miR-181b regulated genes related to cell growth and cell death.
(0.14 MB DOC)

Table S3 The functional annotation clusters related to cell growth and cell death in miR-34a regulated genes.
(0.46 MB DOC)

Table S4 miR-34a regulated genes related to cell growth and cell death.
(0.11 MB DOC)

Table S5 miRNA and siRNAs used for the present study.
(0.03 MB DOC)

Table S6 Primers used for the present study.
(0.03 MB DOC)

## References

[1] Baek D, Villen J, Shin C, Camargo FD, Gygi SP (2008). The impact of microRNAs on protein output.. Nature.

[2] Selbach M, Schwanhausser B, Thierfelder N, Fang Z, Khanin R (2008). Widespread changes in protein synthesis induced by microRNAs.. Nature.

[3] Bartel DP (2009). MicroRNAs: target recognition and regulatory functions.. Cell.

[4] Zhang Y, Lv Q Comparative Analysis of miRNA-mediated Gene Regulation in mammals.. ENCYCLOPEDIA OF LIFE SCIENCES.

[5] Karp X, Ambros V (2005). Developmental biology. Encountering microRNAs in cell fate signaling.. Science.

[6] Wang Y, Blelloch R (2009). Cell cycle regulation by MicroRNAs in embryonic stem cells.. Cancer Res.

[7] Xu P, Guo M, Hay BA (2004). MicroRNAs and the regulation of cell death.. Trends Genet.

[8] Ambros V (2004). The functions of animal microRNAs.. Nature.

[9] Shan SW, Lee DY, Deng Z, Shatseva T, Jeyapalan Z (2009). MicroRNA MiR-17 retards tissue growth and represses fibronectin expression.. Nat Cell Biol.

[10] Kahai S, Lee SC, Lee DY, Yang J, Li M (2009). MicroRNA miR-378 regulates nephronectin expression modulating osteoblast differentiation by targeting GalNT-7.. PLoS One 21;.

[11] Fontana L, Pelosi E, Greco P, Racanicchi S, Testa U (2007). MicroRNAs 17-5p-20a-106a control monocytopoiesis through AML1 targeting and M-CSF receptor upregulation.. Nat Cell Biol.

[12] Garzon R, Calin GA, Croce CM (2009). MicroRNAs in Cancer.. Annu Rev Med.

[13] Hayashita Y, Osada H, Tatematsu Y, Yamada H, Yanagisawa K (2005). A polycistronic microRNA cluster, miR-17-92, is overexpressed in human lung cancers and enhances cell proliferation.. Cancer Res.

[14] Hua Z, Lv Q, Ye W, Wong CK, Cai G (2006). MiRNA-directed regulation of VEGF and other angiogenic factors under hypoxia.. PLoS One.

[15] Nilsen TW (2007). Mechanisms of microRNA-mediated gene regulation in animal cells.. Trends Genet.

[16] Place RF, Li L, Pookot D, Noonan EJ, Dahiya R (2008). MicroRNA-373 induces expression of genes with complementary promoter sequences.. Proc Natl Acad Sci U S A.

[17] Kim DH, Sætrom P, Snøve O, Rossi JJ (2008). MicroRNA-directed transcriptional gene silencing in mammalian cells.. Proc Natl Acad Sci U S A.

[18] Eiring AM, Harb JG, Neviani P, Garton C, Oaks JJ (2010). miR-328 functions as an RNA decoy to modulate hnRNP E2 regulation of mRNA translation in leukemic blasts.. Cell.

[19] Krek A, Grun D, Poy MN, Wolf R, Rosenberg L (2005). Combinatorial microRNA target predictions.. Nat Genet.

[20] Lim LP, Lau NC, Garrett-Engele P, Grimson A, Schelter JM (2005). Microarray analysis shows that some microRNAs downregulate large numbers of target mRNAs.. Nature.

[21] Grimson A, Farh KK, Johnston WK, Garrett-Engele P, Lim LP (2007). MicroRNA targeting specificity in mammals: determinants beyond seed pairing.. Mol Cell.

[22] Bartel DP, Chen CZ (2004). Micromanagers of gene expression: the potentially widespread influence of metazoan microRNAs.. Nat Rev Genet.

[23] Seitz H (2009). Redefining microRNA targets.. Curr Biol.

[24] Tazawa H, Tsuchiya N, Izumiya M, Nakagama H (2007). Tumor-suppressive miR-34a induces senescence-like growth arrest through modulation of the E2F pathway in human colon cancer cells.. Proc Natl Acad Sci U S A.

[25] Huang da W, Sherman BT, Lempicki RA (2009). Systematic and integrative analysis of large gene lists using DAVID bioinformatics resources.. Nat Protoc.

[26] Phimphilai M, Zhao Z, Boules H, Roca H, Franceschi RT (2006). BMP signaling is required for RUNX2-dependent induction of the osteoblast phenotype.. J Bone Miner Res.

[27] Shockley KR, Lazarenko OP, Czernik PJ, Rosen CJ, Churchill GA (2009). PPARgamma2 nuclear receptor controls multiple regulatory pathways of osteoblast differentiation from marrow mesenchymal stem cells.. J Cell Biochem.

[28] Jeon MJ, Kim JA, Kwon SH, Kim SW, Park KS (2003). Activation of peroxisome proliferator-activated receptor-gamma inhibits the Runx2-mediated transcription of osteocalcin in osteoblasts.. J Biol Chem.

[29] Lian JB, Stein GS, Javed A, van Wijnen AJ, Stein JL (2006). Networks and hubs for the transcriptional control of osteoblastogenesis.. Rev Endocr Metab Disord.

[30] Gazzerro E, Canalis E (2006). Bone morphogenetic proteins and their antagonists.. Rev Endocr Metab Disord.

[31] Ye W, Lv Q, Wong CK, Hu S, Fu C (2008). The effect of central loops in miRNA:MRE duplexes on the efficiency of miRNA-mediated gene regulation.. PLoS One.

[32] John B, Enright AJ, Aravin A, Tuschl T, Sander C (2004). Human MicroRNA targets.. PLoS Biol.

[33] Lewis BP, Shih IH, Jones-Rhoades MW, Bartel DP, Burge CB (2003). Prediction of mammalian microRNA targets.. Cell.

[34] Pennisi DJ, Wilkinson L, Kolle G, Sohaskey ML, Gillinder K (2007). Crim1KST264/KST264 mice display a disruption of the Crim1 gene resulting in perinatal lethality with defects in multiple organ systems.. Dev Dyn.

[35] Wilkinson L, Kolle G, Wen D, Piper M, Scott J (2003). CRIM1 regulates the rate of processing and delivery of bone morphogenetic proteins to the cell surface.. J Biol Chem.

[36] Chen J, Bush JO, Ovitt CE, Lan Y, Jiang R (2007). The TGF-beta pseudoreceptor gene Bambi is dispensable for mouse embryonic development and postnatal survival.. Genesis.

