# Supplementary material for: miRNA-Mediated Functional Changes through Co-Regulating Function Related Genes
Source: PLoS One. 2010 Oct 22;5(10):e13558. doi: 10.1371/journal.pone.0013558 (PMC2962631; doi:10.1371/journal.pone.0013558)
Supplement: Table S2 — miR-181b regulated genes related to cell growth and cell death. (0.14 MB DOC) [file pone.0013558.s002.doc]

Table S2. miR-181b regulated genes related to cell growth and cell death.

| **Accession no.** | **Gene**  **symbol** | **Fold**  **change**  **(log2)** | **effect**  **on cell** | **Accession no.** | **Gene**  **symbol** | **Fold change (log2)** | **effect**  **on cell** |
| --- | --- | --- | --- | --- | --- | --- | --- |
| NM_001037165 | FOXK1 | -1.485 | P | NM_018715 | RCC2 | 0.202 | P |
| NM_145012 | CCNY | -0.919 | P | NM_182649 | PCNA | 0.203 | P |
| NM_019003 | SPIN2A | -0.760 | P | NM_013277 | RACGAP1 | 0.203 | P |
| NM_033360 | KRAS | -0.714 | P | NM_004741 | NOLC1 | 0.207 | P |
| NM_016478 | ZC3HC1 | -0.603 | P | NM_031266 | HNRPAB | 0.207 | P |
| NM_006185 | NUMA1 | -0.488 | P | NM_001067 | TOP2A | 0.210 | P |
| NM_001923 | DDB1 | -0.487 | P | NM_003707 | RUVBL1 | 0.210 | P |
| NM_199193 | BRE | -0.482 | P | NM_014303 | PES1 | 0.220 | P |
| NM_001150 | ANPEP | -0.471 | P | NM_000474 | TWIST1 | 0.224 | P |
| NM_014891 | PDAP1 | -0.464 | P | NM_199185 | NPM1 | 0.225 | P |
| NM_002911 | UPF1 | -0.447 | P | NM_003900 | SQSTM1 | 0.231 | P |
| NM_006339 | HMG20B | -0.414 | P | NM_006930 | SKP1 | 0.240 | P |
| NM_007146 | VEZF1 | -0.412 | P | NM_006430 | CCT4 | 0.245 | P |
| NM_016073 | HDGFRP3 | -0.411 | P | NM_001958 | EEF1A2 | 0.246 | P |
| NM_005736 | ACTR1A | -0.378 | P | NM_005180 | BMI1 | 0.254 | P |
| NM_002358 | MAD2L1 | -0.377 | P | NM_001015878 | AURKC | 0.257 | P |
| NM_078471 | MYO18A | -0.372 | P | NM_014624 | S100A6 | 0.270 | P |
| NM_003589 | CUL4A | -0.371 | P | NM_014060 | MCTS1 | 0.298 | P |
| NM_004516 | ILF3 | -0.351 | P | NM_004217 | AURKB | 0.313 | P |
| NM_134442 | CREB1 | -0.347 | P | NM_003592 | CUL1 | 0.327 | P |
| NM_006739 | MCM5 | -0.337 | P | NM_030751 | ZEB1 | 0.333 | P |
| NM_000057 | BLM | -0.336 | P | NM_001261 | CDK9 | 0.346 | P |
| NM_002524 | NRAS | -0.334 | P | NM_002417 | MKI67 | 0.350 | P |
| NM_004526 | MCM2 | -0.308 | P | NM_031966 | CCNB1 | 0.352 | P |
| NM_002388 | MCM3 | -0.288 | P | NM_001316 | CSE1L | 0.368 | P |
| NM_005915 | MCM6 | -0.283 | P | NM_003295 | TPT1 | 0.421 | P |
| NM_002128 | HMGB1 | -0.275 | P | NM_003311 | PHLDA2 | 0.430 | P |
| NM_005982 | SIX1 | -0.274 | P | NM_138555 | KIF23 | 0.434 | P |
| NM_000526 | KRT14 | -0.267 | P | NM_019554 | S100A4 | 0.449 | P |
| NM_005916 | MCM7 | -0.257 | P | NM_181696 | PRDX1 | 0.456 | P |
| NM_002070 | GNAI2 | -0.255 | P | NM_001755 | CBFB | 0.466 | P |
| NM_003858 | CCNK | -0.223 | P | NM_001540 | HSPB1 | 0.501 | P |
| NM_004344 | CETN2 | -0.220 | P | NM_001798 | CDK2 | 0.505 | P |
| NM_013943 | CLIC4 | -0.218 | P | NM_000700 | ANXA1 | 0.510 | P |
|  |  |  |  | NM_000189 | HK2 | 0.525 | P |
|  |  |  |  | NM_005983 | SKP2 | 0.546 | P |
|  |  |  |  | NM_005030 | PLK1 | 0.561 | P |
|  |  |  |  | NM_003286 | TOP1 | 0.632 | P |
|  |  |  |  | NM_002812 | PSMD8 | 0.662 | P |
|  |  |  |  | NM_003591 | CUL2 | 0.803 | P |
|  |  |  |  | NM_004786 | TXNL1 | 1.011 | P |
|  |  |  |  | NM_005496 | SMC4 | 1.011 | P |
|  |  |  |  |  |  |  |  |
|  |  |  |  |  |  |  |  |
| NM_004282 | BAG2 | -0.874 | N | NM_003374 | VDAC1 | 0.210 | N |
| NM_000358 | TGFBI | -0.689 | N | NM_133480 | TADA3L | 0.234 | N |
| NM_001753 | CAV1 | -0.601 | N | NM_152896 | UHRF2 | 0.293 | N |
| NM_003111 | SP3 | -0.559 | N | NM_018947 | CYCS | 0.351 | N |
| NM_004343 | CALR | -0.497 | N | NM_032195 | SON | 0.383 | N |
| NM_002893 | RBBP7 | -0.488 | N | NM_005146 | SART1 | 0.387 | N |
| NM_001553 | IGFBP7 | -0.486 | N | NM_000077 | CDKN2A | 0.531 | N |
| NM_001013836 | MAD1L1 | -0.466 | N | NM_002808 | PSMD2 | 0.731 | N |
| NM_002583 | PAWR | -0.460 | N |  |  |  |  |
| NM_006565 | CTCF | -0.454 | N |  |  |  |  |
| NM_138923 | TAF1 | -0.411 | N |  |  |  |  |
| NM_004551 | NDUFS3 | -0.406 | N |  |  |  |  |
| NM_001425 | EMP3 | -0.397 | N |  |  |  |  |
| NM_003070 | SMARCA2 | -0.396 | N |  |  |  |  |
| NM_005909 | MAP1B | -0.395 | N |  |  |  |  |
| NM_002634 | PHB | -0.379 | N |  |  |  |  |
| NM_016077 | PTRH2 | -0.319 | N |  |  |  |  |
| NM_033081 | DIDO1 | -0.253 | N |  |  |  |  |
| NM_005531 | IFI16 | -0.245 | N |  |  |  |  |
| NM_002759 | EIF2AK2 | -0.228 | N |  |  |  |  |
| NM_201997 | SF1 | -0.224 | N |  |  |  |  |
| NM_001604 | PAX6 | -0.219 | N |  |  |  |  |
| NM_002305 | LGALS1 | -0.212 | N |  |  |  |  |
| NM_022037 | TIA1 | -0.210 | N |  |  |  |  |
| NM_006717 | SPIN1 | -0.208 | N |  |  |  |  |
|  |  |  |  |  |  |  |  |
|  |  |  |  |  |  |  |  |
| NM_005147 | DNAJA3 | -0.523 | D | NM_001014445 | NLE1 | 0.205 | D |
| NM_000414 | HSD17B4 | -0.807 | D | NM_003969 | UBE2M | 0.206 | D |
| NM_006288 | THY1 | -0.558 | D | NM_012138 | AATF | 0.259 | D |
| NM_003078 | SMARCD3 | -0.553 | D | NM_005008 | NHP2L1 | 0.268 | D |
| NM_024348 | DCTN3 | -0.544 | D | NM_004335 | BST2 | 0.287 | D |
| NM_020388 | DST | -0.526 | D | NM_006341 | MAD2L2 | 0.302 | D |
| NM_006088 | TUBB2C | -0.517 | D | NM_012341 | GTPBP4 | 0.311 | D |
| NM_018063 | HELLS | -0.499 | D | NM_014225 | PPP2R1A | 0.339 | D |
| NM_001312 | CRIP2 | -0.491 | D | NM_005858 | AKAP8 | 0.343 | D |
| NM_001321 | CSRP2 | -0.488 | D | NM_025202 | EFHD1 | 0.352 | D |
| NM_004734 | DCLK1 | -0.482 | D | NM_199440 | HSPD1 | 0.430 | D |
| NM_002513 | NME3 | -0.441 | D | NM_018151 | RIF1 | 0.466 | D |
| NM_002705 | PPL | -0.418 | D | NM_015046 | SETX | 0.542 | D |
| NM_018388 | MBNL3 | -0.389 | D | NM_002805 | PSMC5 | 0.704 | D |
| NM_207297 | MBNL1 | -0.317 | D | NM_003088 | FSCN1 | 0.729 | D |
| NM_032727 | INA | -0.304 | D | NM_175932 | PSMD13 | 0.754 | D |
| NM_033133 | CNP | -0.300 | D | NM_000423 | KRT2 | 1.937 | D |
| NM_153200 | EDF1 | -0.285 | D |  |  |  |  |
| NM_003144 | SSR1 | -0.252 | D |  |  |  |  |
| NM_005316 | GTF2H1 | -0.241 | D |  |  |  |  |
| NM_000269 | NME1 | -0.241 | D |  |  |  |  |
| NM_014885 | ANAPC10 | -0.236 | D |  |  |  |  |
| NM_005451 | PDLIM7 | -0.229 | D |  |  |  |  |
| NM_002311 | LIG3 | -0.222 | D |  |  |  |  |
| NM_080738 | EDARADD | -0.220 | D |  |  |  |  |
| NM_001103 | ACTN2 | -0.206 | D |  |  |  |  |
| NM_003998 | NFKB1 | -0.201 | D |  |  |  |  |

P: stimulating cell growth or inhibiting cell death;

N: inhibiting cell growth or enhancing cell death;

D: dual effects or unclear effects on cell growth or cell death.
