# Supplementary material for: miRNA-Mediated Functional Changes through Co-Regulating Function Related Genes
Source: PLoS One. 2010 Oct 22;5(10):e13558. doi: 10.1371/journal.pone.0013558 (PMC2962631; doi:10.1371/journal.pone.0013558)
Supplement: Table S3 — The functional annotation clusters related to cell growth and cell death in miR-34a regulated genes. (0.46 MB DOC) [file pone.0013558.s003.doc]

Table S3. The functional annotation clusters related to cell growth and cell death in miR-34

regulated genes.
