# Supplementary material for: miRNA-Mediated Functional Changes through Co-Regulating Function Related Genes
Source: PLoS One. 2010 Oct 22;5(10):e13558. doi: 10.1371/journal.pone.0013558 (PMC2962631; doi:10.1371/journal.pone.0013558)
Supplement: Table S4 — miR-34a regulated genes related to cell growth and cell death. (0.11 MB DOC) [file pone.0013558.s004.doc]

Table S4. miR-34a regulated genes related to cell growth and cell death.

| Accession no. | Gene  symbol | Fold  change  (log2) | | effect  on cell | Accession no. | | Gene  symbol | | Fold  change  (log2) | | effect  on cell |
| --- | --- | --- | --- | --- | --- | --- | --- | --- | --- | --- | --- |
| M_001216 | CA9 | -3.928 | P | | NM_001554 | CYR61 | | 1.002 | | P | |
| NM_133334 | WHSC1 | -1.971 | P | | NM_058246 | DNAJB6 | | 1.016 | | P | |
| BC011498 | HDAC6 | -1.911 | P | | NM_203330 | CD59 | | 1.019 | | P | |
| NM_022145 | FKSG14 | -1.694 | P | | NM_022873 | G1P3 | | 1.023 | | P | |
| NM_005225 | E2F1 | -1.617 | P | | NM_006460 | HEXIM1 | | 1.135 | | P | |
| NM_002388 | MCM3 | -1.565 | P | | NM_001204 | BMPR2 | | 1.146 | | P | |
| NM_012278 | ITGB1BP2 | -1.527 | P | | NM_012249 | RHOQ | | 1.170 | | P | |
| NM_003579 | RAD54L | -1.515 | P | | NM_004995 | MMP14 | | 1.221 | | P | |
| NM_020242 | KIF15 | -1.510 | P | | NM_004073 | PLK3 | | 1.268 | | P | |
| NM_001018115 | FANCD2 | -1.462 | P | | NM_003840 | TNFRSF10D | | 1.277 | | P | |
| NM_005375 | MYB | -1.415 | P | | NM_014780 | CUL7 | | 1.299 | | P | |
| NM_001790 | CDC25C | -1.351 | P | | NM_002312 | LIG4 | | 1.389 | | P | |
| NM_133265 | AMOT | -1.340 | P | | NM_004290 | RNF14 | | 1.491 | | P | |
| NM_001012409 | SGOL1 | -1.269 | P | | NM_003588 | CUL4B | | 1.523 | | P | |
| NM_022034 | CUZD1 | -1.269 | P | | NM_014755 | SERTAD2 | | 1.535 | | P | |
| NM_080668 | CDCA5 | -1.255 | P | | NM_001206 | KLF9 | | 1.617 | | P | |
| NM_182513 | SPBC24 | -1.238 | P | | NM_005333 | HCCS | | 1.628 | | P | |
| NM_016343 | CENPF | -1.234 | P | | NM_001901 | CTGF | | 1.675 | | P | |
| NM_013349 | NENF | -1.194 | P | | NM_199203 | [TMEM189-UBE2V1](http://bioinfo.weizmann.ac.il/cards-bin/carddisp?TMEM189-UBE2V1) | | 2.092 | | P | |
| NM_016948 | PARD6A | -1.184 | P | | NM_198951 | TGM2 | | 2.259 | | P | |
| NM_133627 | RAD51L3 | -1.171 | P | |  |  | |  | |  | |
| BC004449 | [MEF2B](http://bioinfo.weizmann.ac.il/cards-bin/carddisp?MEF2B) | -1.146 | P | |  |  | |  | |  | |
| NM_004091 | E2F2 | -1.105 | P | |  |  | |  | |  | |
| NM_005342 | HMGB3 | -1.105 | P | |  |  | |  | |  | |
| NM_006306 | SMC1L1 | -1.089 | P | |  |  | |  | |  | |
| NM_001012271 | BIRC5 | -1.080 | P | |  |  | |  | |  | |
| NM_004536 | BIRC1 | -1.074 | P | |  |  | |  | |  | |
| NM_002417 | MKI67 | -1.053 | P | |  |  | |  | |  | |
| NM_005079 | TPD52 | -1.044 | P | |  |  | |  | |  | |
| NM_007109 | TCF19 | -1.029 | P | |  |  | |  | |  | |
| NM_000075 | CDK4 | -1.003 | P | |  |  | |  | |  | |
|  |  |  |  | |  |  | |  | |  | |
|  |  |  |  | |  |  | |  | |  | |
| BE165955 | [DMTF1](http://bioinfo.weizmann.ac.il/cards-bin/carddisp?DMTF1) | -1.761 | N | | NM_003641 | IFITM1 | | 1.035 | | N | |
| NM_015895 | GMNN | -1.544 | N | | NM_181699 | PPP2R1B | | 1.048 | | N | |
| NM_018136 | ASPM | -1.474 | N | | NM_016291 | IHPK2 | | 1.058 | | N | |
| NM_080797 | DATF1 | -1.396 | N | | NM_000400 | ERCC2 | | 1.064 | | N | |
| NM_012177 | FBXO5 | -1.377 | N | | AK125150 | MAPK8 | | 1.065 | | N | |
| NM_015051 | TXNDC4 | -1.362 | N | | NM_005072 | SLC12A4 | | 1.091 | | N | |
| NM_016446 | C9orf127 | -1.304 | N | | NM_003842 | TNFRSF10B | | 1.112 | | N | |
| NM_001024858 | SPTB | -1.241 | N | | NM_015589 | SAMD4 | | 1.264 | | N | |
| NM_031965 | GSG2 | -1.184 | N | | NM_004628 | XPC | | 1.348 | | N | |
| NM_001262 | CDKN2C | -1.047 | N | | NM_013370 | IKL38 | | 1.354 | | N | |
| NM_031215 | CABLES2 | -1.044 | N | | NM_003482 | MLL2 | | 1.375 | | N | |
| NM_016426 | GTSE1 | -1.014 | N | | NM_153687 | IKIP | | 1.484 | | N | |
| NM_024055 | SLC30A5 | -1.014 | N | | NM_012257 | HBP1 | | 1.615 | | N | |
|  |  |  |  | | NM_203339 | CLU | | 1.618 | | N | |
|  |  |  |  | | NM_014454 | SESN1 | | 1.676 | | N | |
|  |  |  |  | | NM_001183 | ATP6AP1 | | 1.823 | | N | |
|  |  |  |  | | NM_080489 | SDCBP2 | | 1.869 | | N | |
|  |  |  |  | | NM_014417 | BBC3 | | 2.113 | | N | |
|  |  |  |  | | NM_014622 | LOH11CR2A | | 2.124 | | N | |
|  |  |  |  | | NM_078467 | CDKN1A | | 3.708 | | N | |
|  |  |  |  | | NM_033285 | TP53NP1 | | 3.846 | | N | |
|  |  |  |  | |  |  | |  | |  | |
|  |  |  |  | |  |  | |  | |  | |
| NM_002466 | MYBL2 | -1.221 | D | | NM_013943 | CLIC4 | | 1.016 | | D | |
| NM_006439 | MAB21L2 | -2.059 | D | | NM_012323 | MAFF | | 1.041 | | D | |
| BC032755 | PEX13 | -2.012 | D | | NM_002517 | NPAS1 | | 1.060 | | D | |
| NM_145659 | IL27 | -1.742 | D | | NM_007054 | KIF3A | | 1.074 | | D | |
| NM_004629 | FANCG | -1.737 | D | | NM_183001 | SHC1 | | 1.176 | | D | |
| NM_000825 | FNRH1 | -1.621 | D | | NM_175605 | TTC10 | | 1.254 | | D | |
| NM_002702 | POU6F1 | -1.326 | D | | BC015514 | TFPI | | 1.283 | | D | |
| NM_006186 | NR4A2 | -1.300 | D | | NM_000346 | SOX9 | | 1.287 | | D | |
| NM_024323 | MGC11271 | -1.234 | D | | NM_002178 | IGFBP6 | | 1.301 | | D | |
| NM_020428 | CTL2 | -1.234 | D | | BM982926 | NPTN | | 1.318 | | D | |
| NM_015341 | BRRN1 | -1.188 | D | | NM_007315 | STAT1 | | 1.331 | | D | |
| NM_016424 | CROP | -1.152 | D | | NM_003879 | CRLAR | | 1.369 | | D | |
| BX640923 | MDM4 | -1.136 | D | | NM_145637 | APOL2 | | 1.372 | | D | |
| NM_012447 | STAG3 | -1.130 | D | | NM_213662 | STAT3 | | 1.397 | | D | |
| NM_018365 | MNS1 | -1.053 | D | | NM_025074 | FRAS1 | | 1.403 | | D | |
|  |  |  |  | | NM_001001522 | TAGLN | | 1.413 | | D | |
|  |  |  |  | | NM_003811 | TNFSF9 | | 1.463 | | D | |
|  |  |  |  | | AL832783 | LMLN | | 1.536 | | D | |
|  |  |  |  | | NM_002505 | NFYA | | 1.868 | | D | |
|  |  |  |  | | NM_080860 | TSGA2 | | 3.241 | | D | |

P: stimulating cell growth or inhibiting apoptosis;

N: inhibiting cell growth or enhancing apoptosis;

D: dual effects or unclear effects on cell growth or apoptosis.
