# Supplementary material for: miRNA-Mediated Functional Changes through Co-Regulating Function Related Genes
Source: PLoS One. 2010 Oct 22;5(10):e13558. doi: 10.1371/journal.pone.0013558 (PMC2962631; doi:10.1371/journal.pone.0013558)
Supplement: Table S5 — miRNA and siRNAs used for the present study. (0.03 MB DOC) [file pone.0013558.s005.doc]

Table S5. miRNA and siRNAs used for the present study.

| miRNA/siRNAs | sequences |
| --- | --- |
| miR-20b  miR-134  miR-504  miR-181b  miR-34a  miR-210  NC ( random sequence )  BAMBI  CRIM1  PPAR_γ_ | 5’CAAAGUGCUCAUAGUGCAGGUAG3’  5’UGUGACUGGUUGACCAGAGGGG3’  5’AGACCCUGGUCUGCACUCUAUC3’  5’AACAUUCAUUGCUGUCGGUGGGU3’  5’UGGCAGUGUCUUAGCUGGUUGU3’  5’CUGUGCGUGUGACAGCGGCUGA3’  sense： 5’UUCUCCGAACGUGUCACGUTT3’  anti-sense：5’ACGUGACACGUUCGGAGAATT3’  sense：5’AUCUGAGCUCAGCGCCUGCTT3’  anti-sense：5’GCAGGCGCUGAGCUCAGAUTT3’  sense：5’GCGGGCGUUUGCGAAGAUGTT3’  anti-sense：5’CAUCUUCGCAAACGCCCGCTT3’  sense：5’GACAUUCCAUUCACAAGAA3’  antisense：5’UUCUUGUGAAUGGAAUGUCTT3’ |
