# Supplementary material for: miRNA-Mediated Functional Changes through Co-Regulating Function Related Genes
Source: PLoS One. 2010 Oct 22;5(10):e13558. doi: 10.1371/journal.pone.0013558 (PMC2962631; doi:10.1371/journal.pone.0013558)
Supplement: Table S6 — Primers used for the present study. (0.03 MB DOC) [file pone.0013558.s006.doc]

Table S6. Primers used for the present study.

| Gene amplified | Primer sequence FOR(5’-3’) | Primer sequence REV(5’-3’) |
| --- | --- | --- |
| BAMBI –WT  BAMBI -MUT  BMP-2  CRIM1 -WT  CRIM1 -MUT  GAPDH  PPARγ-WT  PPARγ-MUT  Runx2 | GACTCTAGATACTGAACAGCTTGAA  GACCGCGTTATCTGAAGACAAAC  GTATCGCAGGCACTCAGGTC  AGCTCTAGATTTGTTGTAGTATGCC  TAATTAACCCATTTGTGCATTGA  TCCATGACAACTTTGGTATCG  TATTCTAGACTTCTTCCAGTTGCACTAT  ATCTTAAAAAGAAAAGGTTTTAGAA  ACTTCCTGTGCTCGGTGCT | CTGACTAGTGTGACAGTGTGTACAA  CTGTCCAGCAGAACTCAAAAGGC  CACTTCCACCACGAATCCAT  TCTACTAGTGTTAAGCATACTGGGT  GTATAAACGAGTTAAGGGCTGTT  TGTAGCCAAATTCGTTGTCA  GTCACTAGTTTCATAATATGGTAATTTT  GATTTTTCACAGTAAATTTCTTAGG  GACGGTTATGGTCAAGGTGAA |
